# Water-Based Perovskite Solar Cells: Precursor Chemistry, Reaction–Diffusion Kinetics, Processing Strategies, and Device Performance

**DOI:** 10.3390/nano16171115

**Published:** 2026-09-04

**Authors:** Zhongjun Dai, Mengnan Li, Yulin Zhang, Xiaofeng He, Jiasheng Chen, Yu Jiao, Qunliang Song

**Affiliations:** 1 School of Materials and Energy Engineering, Xichang University, Xichang 615013, China; leemn1105@163.com (M.L.); chenjs3824@xcc.edu.cn (J.C.); jiaoyu@xcc.edu.cn (Y.J.); 2 Leshan West Silicon Materials Photovoltaic and New Energy Industry Technology Research Institute, Leshan 614000, China; zhangyulin_76rc@wx.lsnu.edu.cn; 3 Institute for Clean Energy and Advanced Materials, School of Materials and Energy, Southwest University, Chongqing 400715, China; hxf666@swu.edu.cn; 4 Yibin Academy of Southwest University, Yibin 644000, China

**Keywords:** aqueous lead precursor, perovskite photovoltaics, sequential deposition, reaction–diffusion kinetics, precursor-film engineering

## Abstract

Water-based perovskite solar cells (W-PSCs) provide a promising route toward reducing the use of hazardous organic solvents during perovskite fabrication. However, their development remains limited by sluggish precursor conversion, incomplete phase transformation, and poor control over film morphology. This review summarizes recent progress in W-PSCs, with particular emphasis on aqueous lead precursors and the subsequent conversion from precursor films to perovskite absorbers. The selection criteria for aqueous lead sources are first discussed in terms of water solubility, anion-Pb^2+^ interactions, precursor-solution stability, and ion-exchange behavior. Thermodynamic and kinetic considerations, including nucleation, crystal growth, reaction–diffusion coupling, and ion transport, are then discussed to provide a framework for understanding the conversion of aqueous precursor films into perovskites. Strategies for improving film formation are further classified into precursor-film and substrate engineering, conversion-process regulation, and ionic/compositional engineering. Particular attention is given to the role of precursor-film microstructure in regulating organic ammonium salt transport and conversion completeness. The photovoltaic performance of regular and inverted W-PSCs is subsequently compared, and the possible origins of their performance differences are discussed from the perspectives of precursor-film formation, perovskite conversion, film morphology, and interfacial properties. Finally, future opportunities in substrate-interface regulation, scalable aqueous processing, precursor and additive design, and life-cycle assessment are outlined. This review provides a reaction-diffusion-based perspective for understanding and improving water-based perovskite photovoltaics.

## 1. Introduction

Perovskite solar cells (PSCs) have attracted considerable commercial interest owing to their low cost, solution-processability, light weight, and flexibility [1]. Their light-absorbing layers are based on metal halide perovskites with an ABX_3_ crystal structure, where A is methylammonium ions (MA^+^), formamidinium ions (FA^+^) and caesium ions (Cs^+^), B is divalent metal cations (Pb^2+^ or Sn^2+^), and X is halide anions (Cl^−^, Br^−^, or I^−^) [2]. The hybrid perovskite materials have numerous advantages, such as a high defect tolerance, superior charge-carrier transport properties, and strong absorption coefficients [3], thereby providing the foundation for the outstanding performance of the PSCs. The power conversion efficiency (PCE) of PSCs has increased from 3.8% in 2009 to approximately 28% [4,5], demonstrating the rapid progress of this photovoltaic technology. Accordingly, research efforts are increasingly extending beyond efficiency optimization toward large-area fabrication, module development, and long-term stability [6,7]. Nevertheless, environmental and health concerns associated with the materials and solvents used during fabrication remain important challenges for the large-scale deployment of PSCs.

Environmental concerns associated with PSC fabrication arise from both the Pb-containing absorber and the solvents used during solution processing. Although the Pb loading in PSCs is relatively low, typically approximately 0.4 g/m^2^ [8], the release of soluble Pb during fabrication, operation, or disposal remains an important environmental concern. The EU Restriction of Hazardous Substances (RoHS) directive limits the Pb content in homogeneous materials to 0.1 wt% [9], highlighting the need for effective Pb containment and recycling strategies. Accordingly, encapsulation and Pb-sequestration approaches have been developed to reduce the risk of Pb leakage from damaged devices [10,11]. In parallel, the extensive use of polar aprotic solvents during perovskite processing represents another important environmental and occupational-health challenge. High-quality perovskite films are commonly deposited from dimethylformamide (DMF), dimethyl sulfoxide (DMSO), γ-butyrolactone (GBL), or N-methylpyrrolidinone (NMP), which are used to dissolve lead halides and organic ammonium salts such as formamidinium iodide (FAI), methylammonium iodide (MAI), and methylammonium chloride (MACl) [12,13,14]. The toxicity and processing characteristics of several of these solvents raise concerns regarding their extensive use in large-scale manufacturing [15,16]. Therefore, reducing the dependence on hazardous organic solvents is an important aspect of the development of more sustainable PSC fabrication technologies.

Developing lower-hazard solvent systems to replace conventional polar aprotic solvents is therefore an important direction for sustainable PSC fabrication. Several alternative solvents, including γ-valerolactone, triethyl phosphate, ethanol, and acetonitrile, have been investigated for perovskite processing [17,18]. Water is particularly attractive because of its abundance, low cost, non-flammability, and low intrinsic toxicity. However, aqueous processing differs fundamentally from conventional organic-solvent processing. In typical organic-solvent systems, solvent coordination, evaporation, and supersaturation strongly influence perovskite nucleation and crystallization [19]. In aqueous systems, water additionally interacts strongly with ionic precursors and can promote hydrolysis or hydration reactions [20,21,22]. Direct exposure of the formed perovskite to water may also destabilize the perovskite lattice and induce decomposition products such as PbI_2_ [23,24]. Consequently, aqueous processing requires precursor chemistries and deposition routes that are distinct from those used in conventional perovskite fabrication.

In this review, W-PSCs primarily refer to devices in which the perovskite is prepared using an aqueous lead precursor, particularly aqueous Pb(NO_3_)_2_, followed by conversion with organic ammonium halides. The term therefore does not imply that all precursor components or functional layers of the device are processed exclusively from water. This review focuses on the physicochemical processes that govern aqueous-precursor-based perovskite fabrication. We first discuss the selection of aqueous lead precursors and the requirements for stable precursor solutions and controllable film deposition. The thermodynamic and kinetic processes involved in the conversion of Pb(NO_3_)_2_ precursor films are then considered, with emphasis on nucleation, crystal growth, ion exchange, and diffusion. Subsequently, representative strategies for improving precursor-film morphology and accelerating perovskite conversion are summarized. The performance of regular and inverted W-PSCs is further compared to identify the factors that currently limit inverted device architectures. Finally, remaining challenges and future research directions for scalable and environmentally responsible water-based PSCs are discussed.

## 2. Fabrication of Water-Based Perovskite Films

### 2.1. Materials Selection

**Figure 1:**
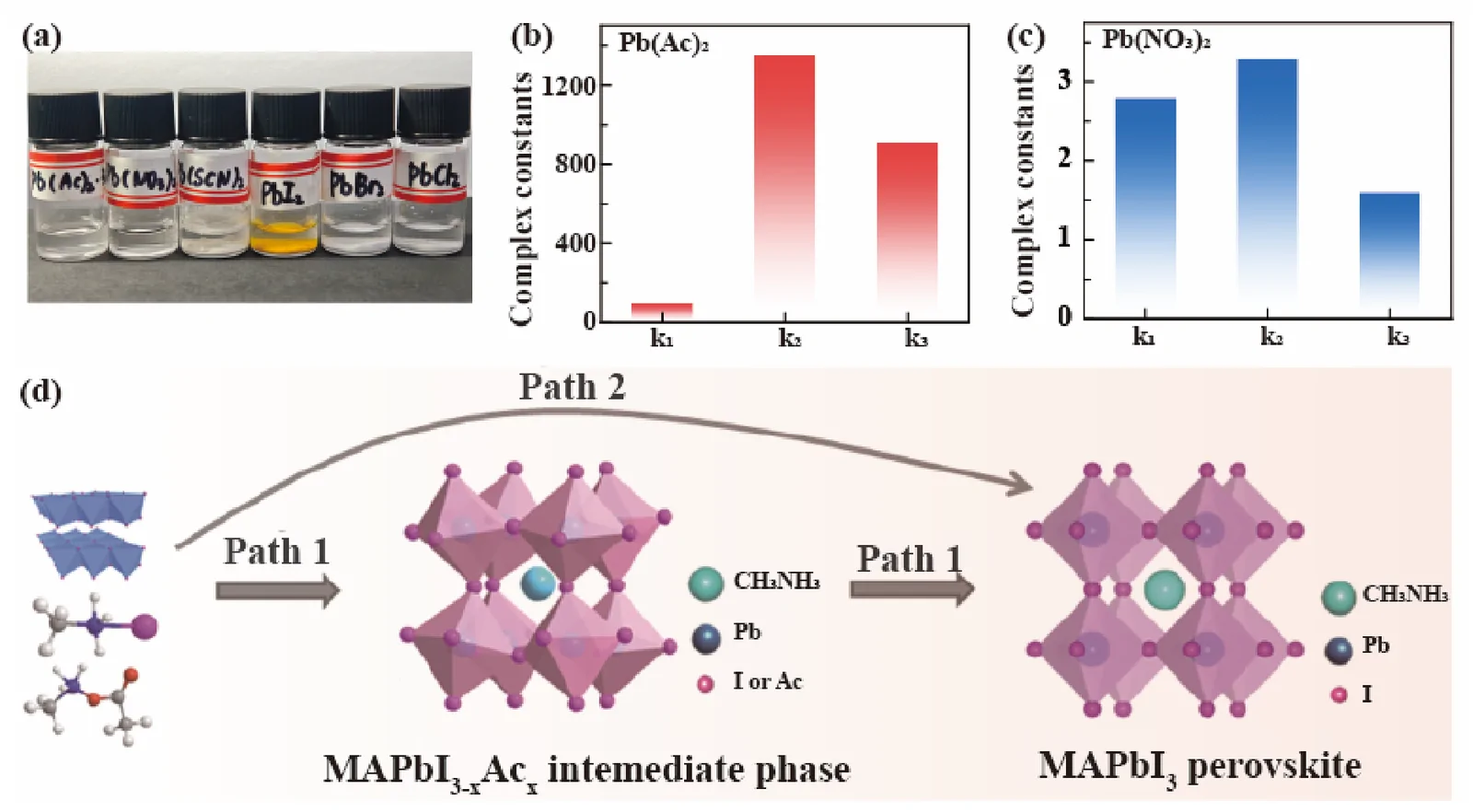
(**a**) Photographs show the solubility of lead salts in water. Stability constants derived for (**b**) Pb(Ac)_2_ and (**c**) Pb(NO_3_)_2_, respectively [34,35]. (**d**) Schematic depiction of acetate involvement in the intermediate-phase formation during perovskite crystallization [29] (Reproduced from ref [29]; Copyright 2017, Royal Society of Chemistry).

A homogeneous and stable precursor solution is a prerequisite for reproducible perovskite-film fabrication. However, many commonly used lead salts exhibit limited solubility in water, as summarized in Figure 1a and Table 1. Pb(Ac)_2_ and Pb(NO_3_)_2_ exhibit substantially higher aqueous solubility than lead halides and therefore represent practical candidates for aqueous precursor preparation. High solubility alone, however, is insufficient for selecting a suitable lead source. The coordination between Pb^2+^ and the counter anion, the stability of the aqueous precursor solution, the subsequent ion-exchange kinetics, and the removal of reaction byproducts must also be considered. Compared with NO_3_^−^, Ac^−^ interacts more strongly with Pb^2+^ [25], as illustrated in Figure 1b,c. Pb(Ac)_2_ can form acetate-containing Pb complexes in aqueous solution, which modifies the availability of Pb^2+^ for subsequent ion-exchange reactions. Pb(Ac)_2_ is also susceptible to hydrolysis under certain aqueous conditions, which may compromise solution stability and film uniformity [26]. During perovskite formation, acetate can participate in intermediate-phase formation and interact with organic cations [27,28,29]. Their subsequent replacement by halide ions introduces an additional compositional transformation during crystallization, as shown in Figure 1d. These characteristics make the conversion chemistry of Pb(Ac)_2_ more complex than that of Pb(NO_3_)_2_.

Efficient ion exchange between the lead precursor and OAS is important for complete perovskite conversion. The transport and removal of precursor anions are influenced not only by their effective ionic size but also by solvation, coordination strength, local composition, and the structure of the precursor film. NO_3_^−^ (~1.96 Å) has a smaller effective ionic radius than Ac^−^ (~2.25 Å) [30,31], which may reduce transport constraints during the conversion process. In addition, the relatively weak interaction between NO_3_^−^ and Pb^2+^ favors ion exchange with halide. Taken together, high aqueous solubility, favorable ion-exchange behavior, and relatively facile removal of nitrate-containing species make Pb(NO_3_)_2_ one of the most practical aqueous lead precursors used for water-based perovskite films. The use of aqueous Pb(NO_3_)_2_ also provides opportunities for managing lead-containing process waste. Residual Pb(NO_3_)_2_ can be recovered from aqueous waste streams or precipitated using suitable chelating agents [32,33]. Nevertheless, aqueous processing does not eliminate the environmental risk associated with Pb. Appropriate collection, recovery, and treatment of Pb-containing wastewater remain necessary for evaluating the environmental advantages of water-based PSC fabrication.

### 2.2. Fabrication Processes

**Figure 2:**
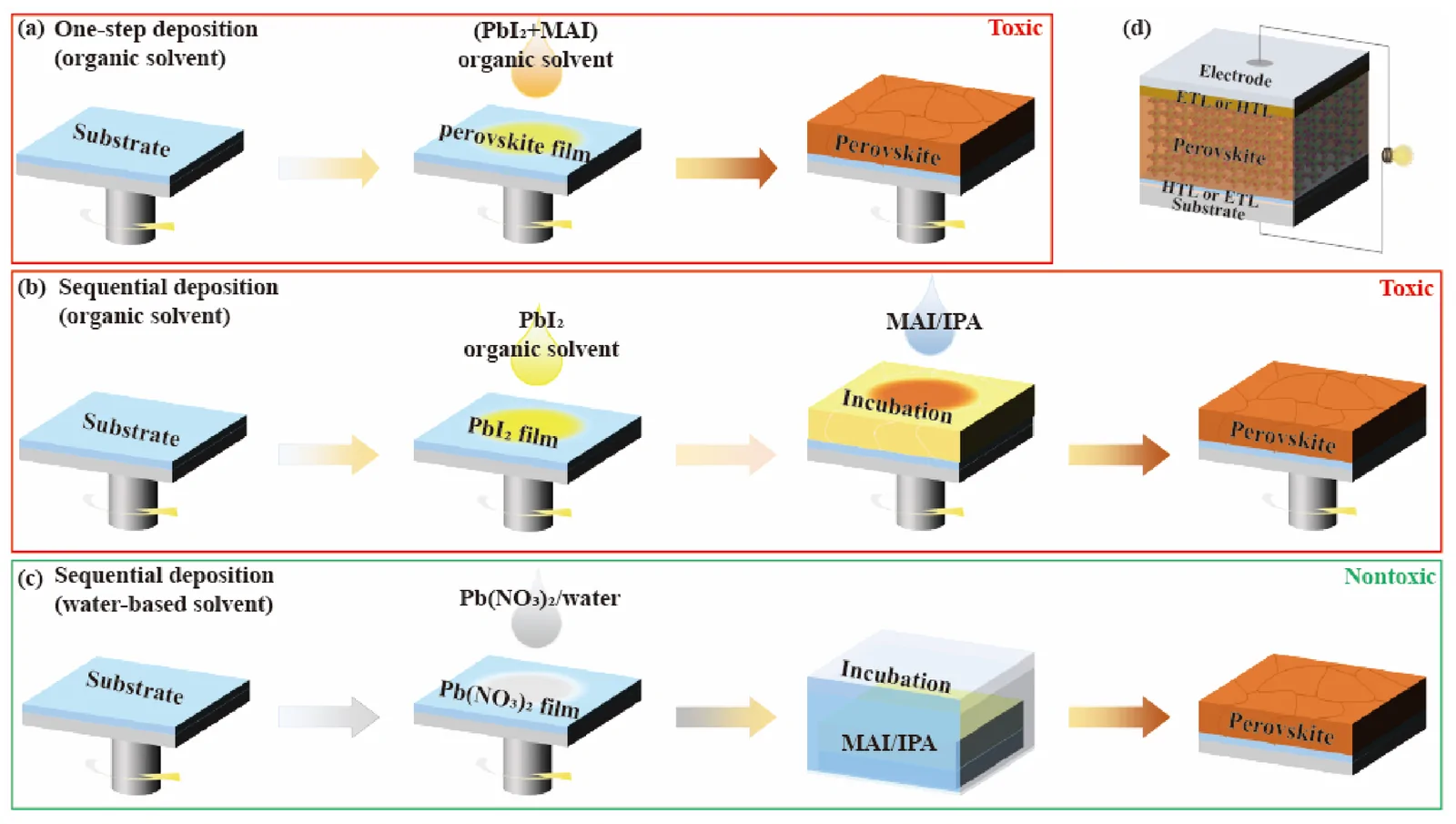
Author-generated schematic illustrations of (**a**) one-step and (**b**) sequential deposition for conventional perovskite films, (**c**) sequential deposition for water-based perovskite films, and (**d**) the general device configuration of PSCs.

Because Pb(NO_3_)_2_ is highly soluble in water whereas organic ammonium halides readily react with Pb^2+^ to form poorly soluble lead halides, directly combining Pb(NO_3_)_2_ and OAS in a single aqueous precursor solution generally causes precipitation. A conventional one-step precursor formulation is therefore difficult to establish for this system, as illustrated schematically in Figure 2a. Sequential deposition provides a practical alternative. In this process, an aqueous Pb(NO_3_)_2_ precursor film is first deposited, followed by exposure to OAS. Ion exchange, interdiffusion, and subsequent perovskite crystallization then convert the precursor film into the perovskite absorber, as illustrated in Figure 2b,c. The resulting perovskite layer can subsequently be integrated with charge-transport layers and electrodes to construct PSCs, as schematically shown in Figure 2d.

**Figure 3:**
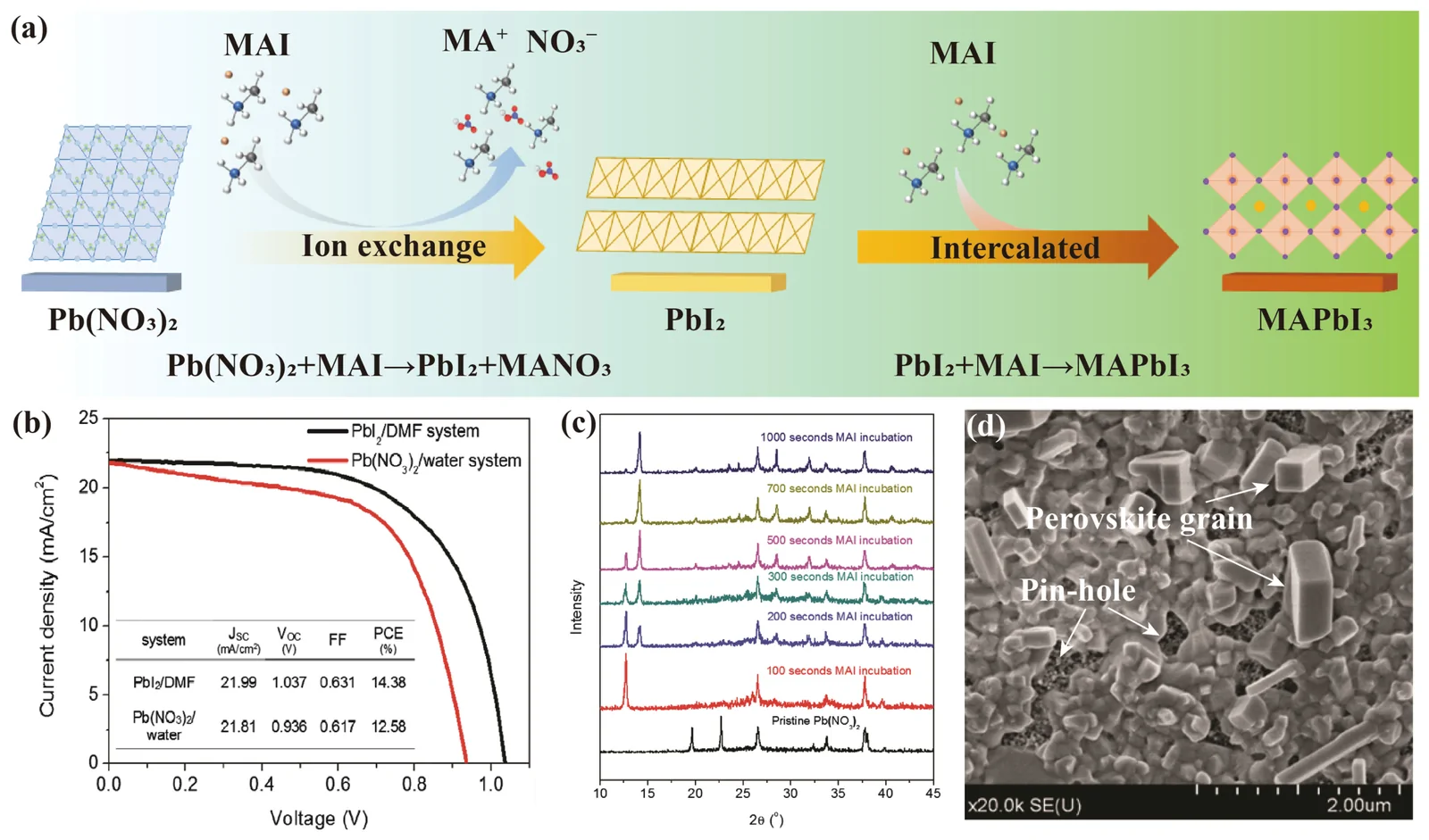
(**a**) Perovskite crystal formation from Pb(NO_3_)_2_ film. (**b**) Current J-V response of solar cells based on water-based vs. conventional perovskite films. (**c**) XRD spectra of Pb(NO_3_)_2_ film incubated in OAS for sequential durations. (**d**) Surface SEM micrograph of the perovskite film derived from the Pb(NO_3_)_2_ film after 1000 s of incubation in an OAS solution [36] (Reproduced from ref. [36]; Copyright 2015, Royal Society of Chemistry).

In 2015, Hsieh et al. applied sequential deposition for the construction of perovskite films derived from a water-based precursor [36]. They deposited the Pb(NO_3_)_2_ film first from an aqueous Pb(NO_3_)_2_ solution, and then immersed the Pb(NO_3_)_2_ film into an OAS solution for perovskite incubation, as shown in Figure 2c. Figure 3a illustrates the conversion route, which proceeds through two successive reactions. Pb(NO_3_)_2_ first undergoes ion exchange with MAI to form PbI_2_ and MANO_3_, after which additional MAI reacts with PbI_2_ to form MAPbI_3_. Using this route, Hsieh et al. obtained W-PSCs with PCEs above 12%, demonstrating the feasibility of aqueous Pb(NO_3_)_2_ as a lead precursor for perovskite fabrication (Figure 3b). Nevertheless, the conversion process from Pb(NO_3_)_2_ to perovskite is notably slow. The reported conversion from the Pb(NO_3_)_2_ precursor film to the perovskite required an incubation time approaching 1000 s under the conditions used in this early study (Figure 3c). The slow conversion kinetics not only led to the formation of noticeable holes within the final films but also induced the formation of abnormally large perovskite grains on the film surface. The resultant rough morphology and porous structure severely degraded film quality (Figure 3d), restricting the performance enhancement of the devices.

In sequential aqueous processing, the Pb(NO_3_)_2_ film serves both as the Pb source and as the structural matrix through which the OAS must diffuse during conversion. Its thickness, grain size, porosity, crystallinity, and interfacial contact with the substrate therefore strongly affect the subsequent reaction. At the same time, inward transport of OAS and outward transport of nitrate-containing species determine the progression of the conversion front through the film. Consequently, the final perovskite morphology cannot be considered independently of precursor-film formation and reaction–diffusion kinetics. Understanding the relationship between precursor microstructure, ion transport, and phase conversion is therefore central to improving water-based perovskite films.

## 3. Thermodynamics and Kinetics of Water-Based Perovskite Fabrication

**Figure 4:**
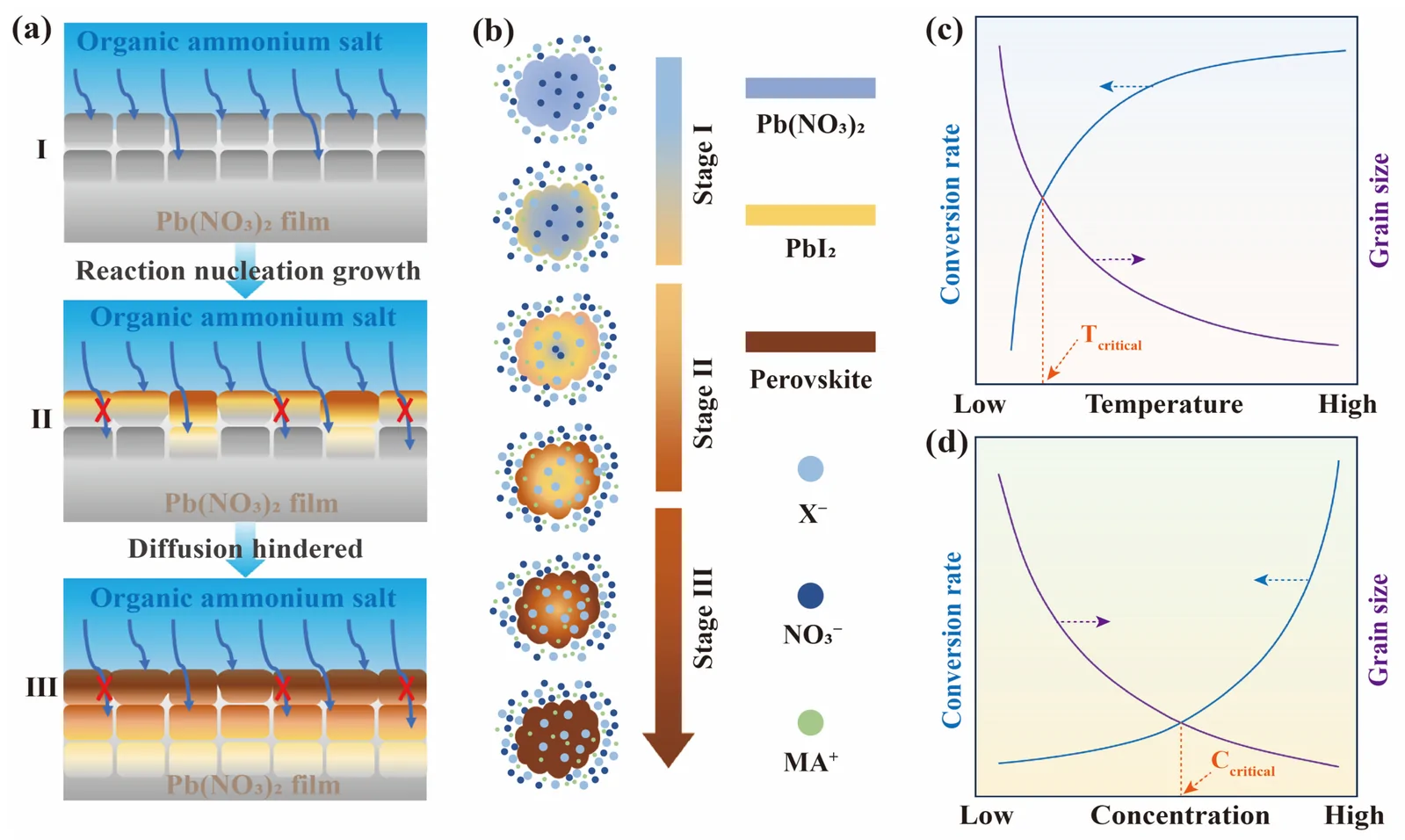
(**a**) Author-generated conceptual illustration of OAS transport through the precursor film accompanied by perovskite nucleation and growth. (**b**) Author-generated schematic illustration of OAS transport toward the interior of Pb(NO_3_)_2_ grains and subsequent perovskite conversion, based on the conversion mechanism reported in Ref. [41]. Author-generated qualitative relationships between conversion behavior and grain size with (**c**) reaction temperature and (**d**) OAS concentration. Panels (**c**,**d**) illustrate qualitative trends and do not represent fitted experimental data. The blue and purple arrows indicate the trends in conversion rate and grain size, respectively.

The conversion of aqueous Pb(NO_3_)_2_ precursor films into perovskite is a multistep process that proceeds from the exposed surface toward the film interior (Figure 4a) and involves coupled chemical reaction and mass transport. Because complete conversion requires sustained OAS transport through the evolving film, prolonged processing can promote grain coarsening and deteriorate film morphology [36]. A kinetic framework that considers interfacial reaction, nucleation, crystal growth, and diffusion is therefore useful for understanding the factors governing precursor conversion and film formation.

The conversion process can be discussed in three overlapping regimes: the initial interfacial reaction, nucleation and propagation of the conversion front, and subsequent conversion with an increasing contribution from mass transport. These regimes are not sharply separated because chemical reaction, nucleation, crystal growth, and ion transport can occur concurrently. Their relative contributions change as the conversion front moves from the exposed surface toward the interior of the precursor film.

Stage 1: Interfacial reaction. When the Pb(NO_3_)_2_ film contacts the OAS solution, ion exchange occurs rapidly near the film/solution interface. Pb(NO_3_)_2_ is converted into PbI_2_-containing species, followed by the formation of the perovskite phase. Previous studies indicate that the initial interfacial reaction is considerably faster than the overall transformation of the entire precursor film [37]. Therefore, the long conversion time observed experimentally is unlikely to originate solely from the intrinsic reaction rate at the exposed surface.

Stage 2: Nucleation and reaction-front propagation. As OAS continues to penetrate into the precursor film, new perovskite nuclei form in regions containing reaction-derived PbI_2_ and OAS. Their subsequent growth contributes to the propagation of the conversion front toward the interior of the film. The transformation behavior can be phenomenologically described using the Johnson–Mehl–Avrami–Kolmogorov (JMAK) model under approximately isothermal conditions [38,39]

(1) $$Y=1-exp(-K_{1}t^{n})$$

where *Y* is the conversion fraction of the perovskite phase, *t* is the conversion time, *n* is the Avrami exponent, and *K*_1_ is the effective rate constant with units of time^−*n*^. The JMAK model describes the time-dependent evolution of the transformed fraction but does not independently identify the rate-controlling process. Because the conversion of Pb(NO_3_)_2_ films involves concurrent ion exchange, nucleation, crystal growth, and ion transport, the fitted parameters should be regarded as effective kinetic descriptors rather than direct measures of a single elementary mechanism [39]. Classical nucleation theory provides a useful thermodynamic basis for describing the formation of a new perovskite phase [40]

(2) $$N=K_{2}exp\left(-\frac{∆G^{*}}{k_{B}T}\right)exp\left(-\frac{Q}{k_{B}T}\right)$$

where *N* represents the nucleation rate per unit volume, *K*_2_ is the corresponding pre-exponential factor, ΔG* is the critical nucleation free-energy barrier, *Q* is the kinetic barrier associated with atomic or ionic rearrangement, *k_B_* is the Boltzmann constant, and T is the absolute temperature. When *k_B_* is used, ΔG* and *Q* are expressed as energies per nucleating entity (*J*), so that the exponential terms are dimensionless. This expression is used here as a classical description of the energetic requirement for nucleation rather than as a quantitative rate equation specifically derived for Pb(NO_3_)_2_ conversion.

In the Pb(NO_3_)_2_ system, perovskite nucleation occurs after local ion exchange and the formation of PbI_2_-containing regions. The nucleation behavior is therefore affected by the local precursor composition, OAS supply, temperature, interfacial environment, and mass transport through the precursor film. Classical nucleation theory is used here to describe the thermodynamic requirement for the formation of stable nuclei rather than to provide a system-specific nucleation-rate expression for the complete Pb(NO_3_)_2_-to-perovskite transformation.

After the initial surface region is converted, the contribution of mass transport to the overall transformation becomes increasingly important. At the beginning of incubation, OAS directly contacts the exposed Pb(NO_3_)_2_ surface, where ion exchange and perovskite formation can proceed rapidly. As the reacted region develops and the conversion front moves toward the film interior, OAS must pass through this region before reaching the remaining precursor. OAS must also penetrate from the exterior toward the interior of individual Pb(NO_3_)_2_ grains, as illustrated in Figure 4a,b. Structural rearrangement and volume expansion during perovskite formation can further reduce the accessibility of deeper precursor regions [42].

The kinetic descriptions introduced above address complementary aspects of this process. The JMAK model describes the time dependence of the transformed fraction but does not independently identify the rate-controlling mechanism. When an effective conversion rate constant is obtained at different temperatures, its temperature dependence can be described using an Arrhenius-type relationship [39]:

(3) $$k_{eff}=A{exp}^{-\frac{E_{a,\;eff}}{RT}}$$

where *k_eff_* is the effective conversion rate constant, *A* is the pre-exponential factor with the same units as *k_eff_*, *E_a_*_,*eff*_ is the apparent activation energy (J/mol), *R* is the gas constant (J/(mol·K)), and *T* is the absolute temperature (*K*). In the present system, *E_a_*_,*eff*_ may include contributions from ion transport, interfacial reaction, and structural rearrangement and should therefore not be assigned exclusively to diffusion.

Mass transport through the evolving precursor film can be described using Fick’s first law [43]:

(4) $$J=-D_{eff}\frac{dC}{dx}$$

where *J* is the OAS flux (mol/(m^2^·s)), *D_eff_* is the effective diffusivity (m^2^/s), *C* is the OAS concentration (mol/m^3^), and *x* is the transport distance (m). Therefore, *dC*/*dx* has units of mol/m^4^ and the resulting flux has units of mol/(m^2^·s). In porous precursor films, *D_eff_* is influenced by pore size, pore connectivity, and transport tortuosity. Smaller precursor grains and interconnected pores can improve OAS access to the film interior, whereas dense reacted regions and poorly connected pores increase transport resistance.

The JMAK, Arrhenius, and Fick descriptions therefore address different aspects of the same conversion process rather than separate sequential mechanisms. As conversion proceeds, the increasing transport distance and resistance make mass transport progressively more important. The later conversion stage is therefore better described as having an increasing diffusion contribution rather than undergoing an abrupt transition from reaction-controlled to diffusion-controlled behavior. The relationships shown in Figure 4c,d should accordingly be regarded as qualitative trends rather than universal quantitative descriptions.

Stage 3: Continued conversion and crystal growth under increasing transport limitation. Once stable perovskite nuclei have formed, the factors governing nucleation should be distinguished from those controlling subsequent crystal growth. According to classical nucleation theory, the critical nucleation barrier determines whether an embryo can develop into a stable nucleus [40]. After stable nuclei are formed, continued growth requires the transport of OAS-derived species to the growth front and their subsequent incorporation into the perovskite lattice. Crystal growth is therefore influenced by both mass transport and interfacial growth kinetics.

During the sequential conversion of Pb(NO_3_)_2_ films, the conditions for crystal growth change continuously. As the conversion front moves toward the film interior, the reacted region becomes progressively thicker and OAS must travel through a longer pathway before reaching the remaining precursor. Structural rearrangement and volume expansion can further modify the available transport pathways. Interfacial incorporation remains important when OAS transport is sufficiently rapid. The later stage is therefore better described as continued conversion and crystal growth under increasing transport limitation rather than as a process controlled purely by diffusion.

Nucleation density can influence the final grain size because it affects the space available for subsequent crystal growth. However, the final grain size in this system is also affected by OAS transport, interfacial growth kinetics, precursor microstructure, and processing time. A simple quantitative relationship between nucleation density and final grain size is therefore not appropriate for describing the multistep conversion of aqueous Pb(NO_3_)_2_ precursor films.

Temperature and OAS concentration can influence both conversion and crystal growth. Increasing temperature can enhance ion mobility and reaction rates, while OAS concentration affects the concentration gradient and transport into the precursor film. Excessively rapid conversion near the exposed surface may restrict subsequent OAS penetration, whereas prolonged treatment can promote grain coarsening or perovskite degradation. Appropriate processing conditions should therefore maintain sufficient OAS transport throughout the precursor film while avoiding excessive crystal growth.

In summary, the transformation of aqueous Pb(NO_3_)_2_ precursor films is governed by coupled ion exchange, nucleation, crystal growth, and mass transport. The initial surface reaction is relatively rapid, whereas subsequent conversion of the film interior increasingly depends on OAS diffusion and the evolving microstructure of the precursor and product layers. This reaction–diffusion framework explains why precursor grain size, pore structure, OAS concentration, and processing temperature strongly influence conversion completeness and final film morphology, and provides a basis for the processing strategies discussed below.

## 4. Optimized Strategies for W-PSCs

To address incomplete conversion and poor morphology in water-based perovskite films, reported strategies can be broadly classified according to the stage of film formation that they primarily regulate. (I) Precursor-film and substrate engineering regulates wettability, nucleation, grain size, and pore structure during Pb(NO_3_)_2_ deposition. (II) Conversion-process and external-field engineering modifies the reaction environment or energy input to accelerate ion transport and phase transformation. (III) Ionic and compositional regulation adjusts the species involved in ion exchange and perovskite formation to improve conversion kinetics and film quality. These approaches are discussed below according to their primary roles in the reaction–diffusion process.

### 4.1. Precursor Film and Substrate Engineering

In water-based perovskite fabrication, the Pb(NO_3_)_2_ precursor film determines the initial structural environment for the subsequent conversion reaction. Its grain size, coverage, porosity, crystallographic characteristics, and interfacial contact with the substrate influence OAS infiltration and progression of the conversion front. Precursor-film formation is in turn governed by the physicochemical properties of the substrate, including wettability, surface energy, roughness, and surface potential, as well as by solution properties such as surface tension and concentration [44]. Regulation of both the substrate and precursor solution is therefore necessary for obtaining a suitable Pb(NO_3_)_2_ film.

**Figure 5:**
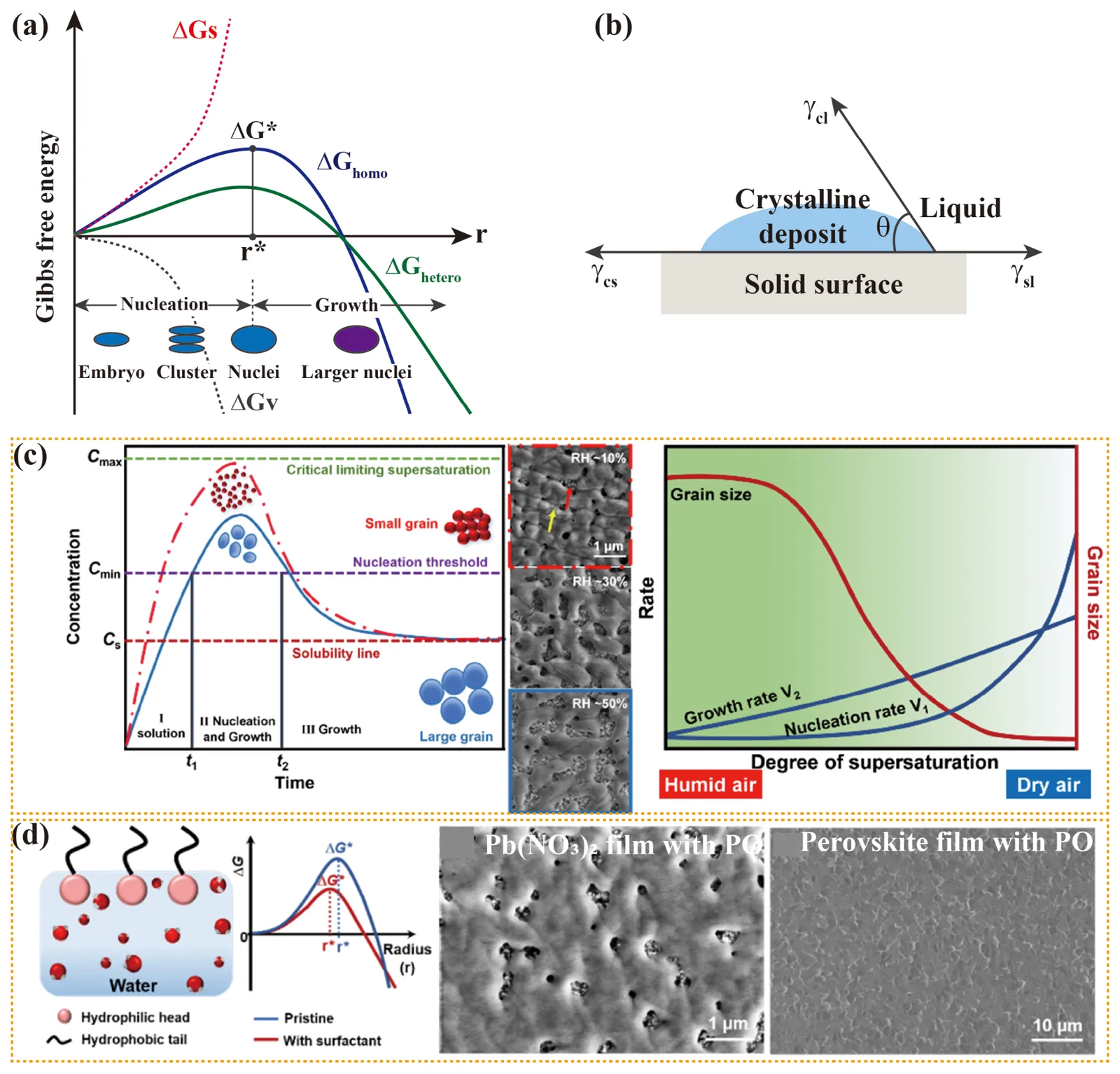
(**a**) Free-energy change vs. nucleus radius in classical homogeneous nucleation. ΔG* and r* denote the critical Gibbs free-energy barrier and critical nucleus radius, respectively. (**b**) Contact angle for heterogeneous nucleation. (**c**) LaMer diagram of the homogeneous nucleation pathway [47]. The red and yellow arrows indicate the mp-TiO_2_ substrate and Pb(NO_3_)_2_ film, respectively. (**d**) PO adsorption layer at the aqueous surface, the corresponding ΔG-r relationship, and SEM micrographs of the Pb(NO_3_)_2_ film and derived perovskite after 2 mg PO addition [47] (reproduced from ref. [47]; Copyright 2024, Royal Society of Chemistry).

**Figure 6:**
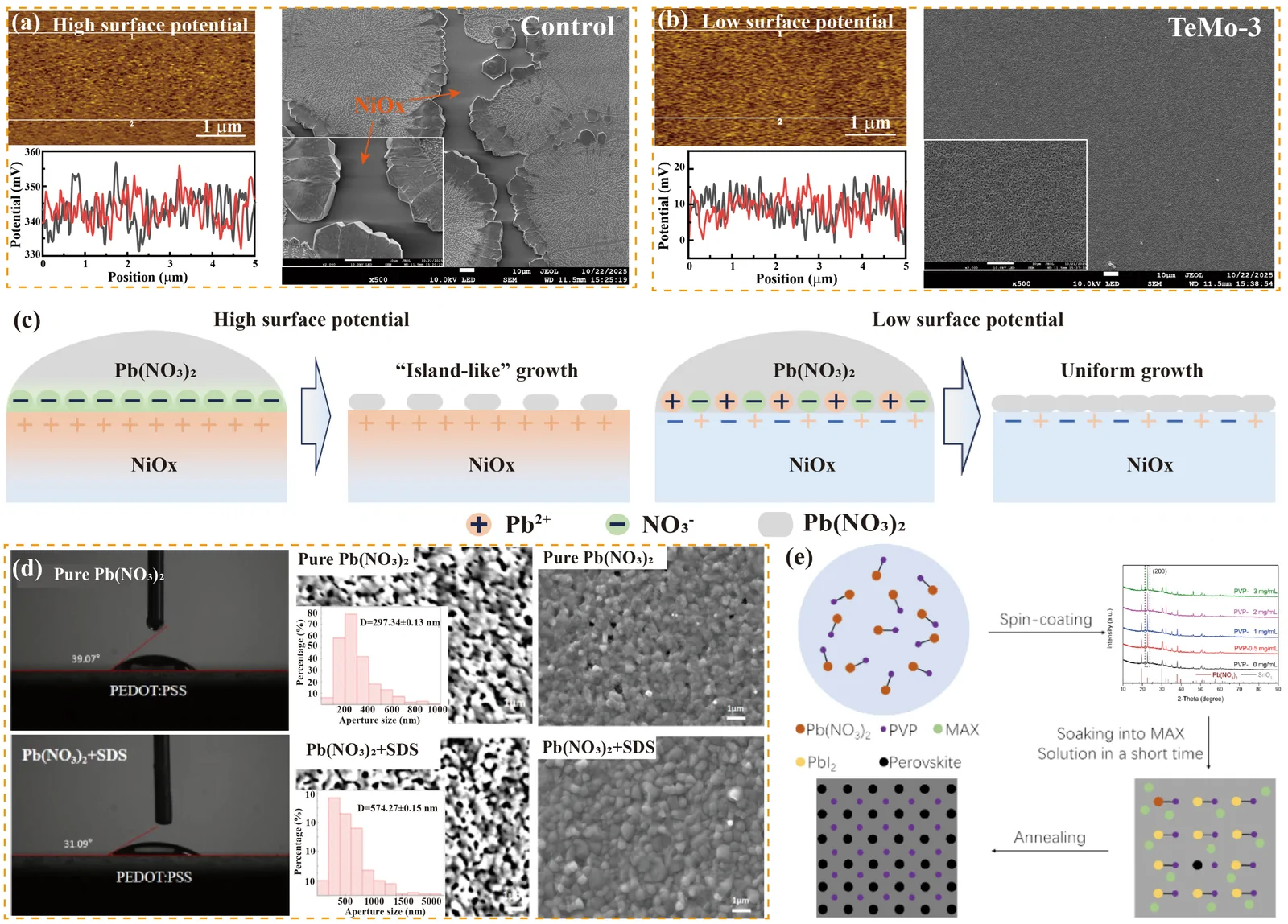
(**a**) Surface potential maps of the control NiO_x_ film and the corresponding SEM images of the Pb(NO_3_)_2_ layer. (**b**) Surface potential maps of the TeMo-3 NiO_x_ film and the corresponding SEM images of the Pb(NO_3_)_2_ layer. (**c**) Influence of substrate surface potential (high vs. low) on the deposition behavior of Pb(NO_3_)_2_ films [44]. (reproduced from ref. [44]; Copyright 2025, American Chemical Society) (**d**) Static contact angles of Pb(NO_3_)_2_ solutions and surface morphologies of the resulting Pb(NO_3_)_2_ and perovskite films on PEDOT:PSS substrates, with and without SDS doping [42] (reproduced from ref. [42]; Copyright 2025, Multidisciplinary Digital Publishing Institute) (**e**) Perovskite film deposition in the presence of PVP, together with XRD patterns of Pb(NO_3_)_2_ films prepared with varying PVP concentrations [48] (reproduced from ref. [48]; Copyright 2019, Beilstein-Institut).

According to classical nucleation theory, heterogeneous nucleation of Pb(NO_3_)_2_ is strongly influenced by interfacial energy and substrate wettability, as illustrated in Figure 5a,b [45]. Feng et al. reduced the surface tension of the aqueous Pb(NO_3_)_2_ solution from 5.6 × 10^−2^ to 2.6 × 10^−2^ N/m by introducing Surfynol 465, which improved solution spreading and promoted the formation of more continuous precursor films [46]. Zhang et al. further demonstrated that precursor-film formation is jointly affected by substrate conditions and solution surface tension [47]. By combining dry-air-assisted deposition with potassium oleate (PO), they regulated Pb(NO_3_)_2_ crystallization and obtained dense and pinhole-free precursor films, leading to a champion device PCE of 24.14% (Figure 5c,d). Dai et al. showed that substrate surface free energy and surface potential influence Pb(NO_3_)_2_ deposition on NiO_x_ (Figure 6a–c) [44]. A temperature-modulated treatment improved the uniformity of the precursor film, enabling the first reported NiO_x_-based planar inverted W-PSCs with a champion PCE of 18.34%.

It is important to distinguish controlled porosity in the precursor layer from morphological defects in the final perovskite film. Interconnected pores or channels within the Pb(NO_3_)_2_ film can provide transport pathways for OAS and promote conversion of the film interior. In contrast, large pinholes, residual voids, or discontinuities remaining after perovskite formation can increase leakage and recombination and are detrimental to device operation. An effective precursor structure should therefore provide sufficient ion-transport pathways without sacrificing film coverage or structural continuity. Zheng et al. incorporated sodium dodecyl sulfonate into the precursor solution [42], which altered Pb(NO_3_)_2_ nucleation and increased the characteristic micropore size from 297.34 to 574.27 nm (Figure 6d). The enlarged pore network facilitated OAS penetration, shortened the conversion time, and improved the morphology and crystallinity of the resulting perovskite film, increasing the device efficiency from 12.27% to 14.82%. Because conversion is initially rapid at the exposed surface and is accompanied by volume expansion, early formation of a dense perovskite layer can restrict further OAS transport into the film interior. Du et al. therefore introduced polyvinylpyrrolidone (PVP) to regulate Pb(NO_3_)_2_ crystallization and its interaction with Pb^2+^ [48]. The resulting Pb-PVP interaction moderated the reaction with MAI and provided additional time for inward OAS diffusion, promoting more uniform perovskite formation (Figure 6e).

**Figure 7:**
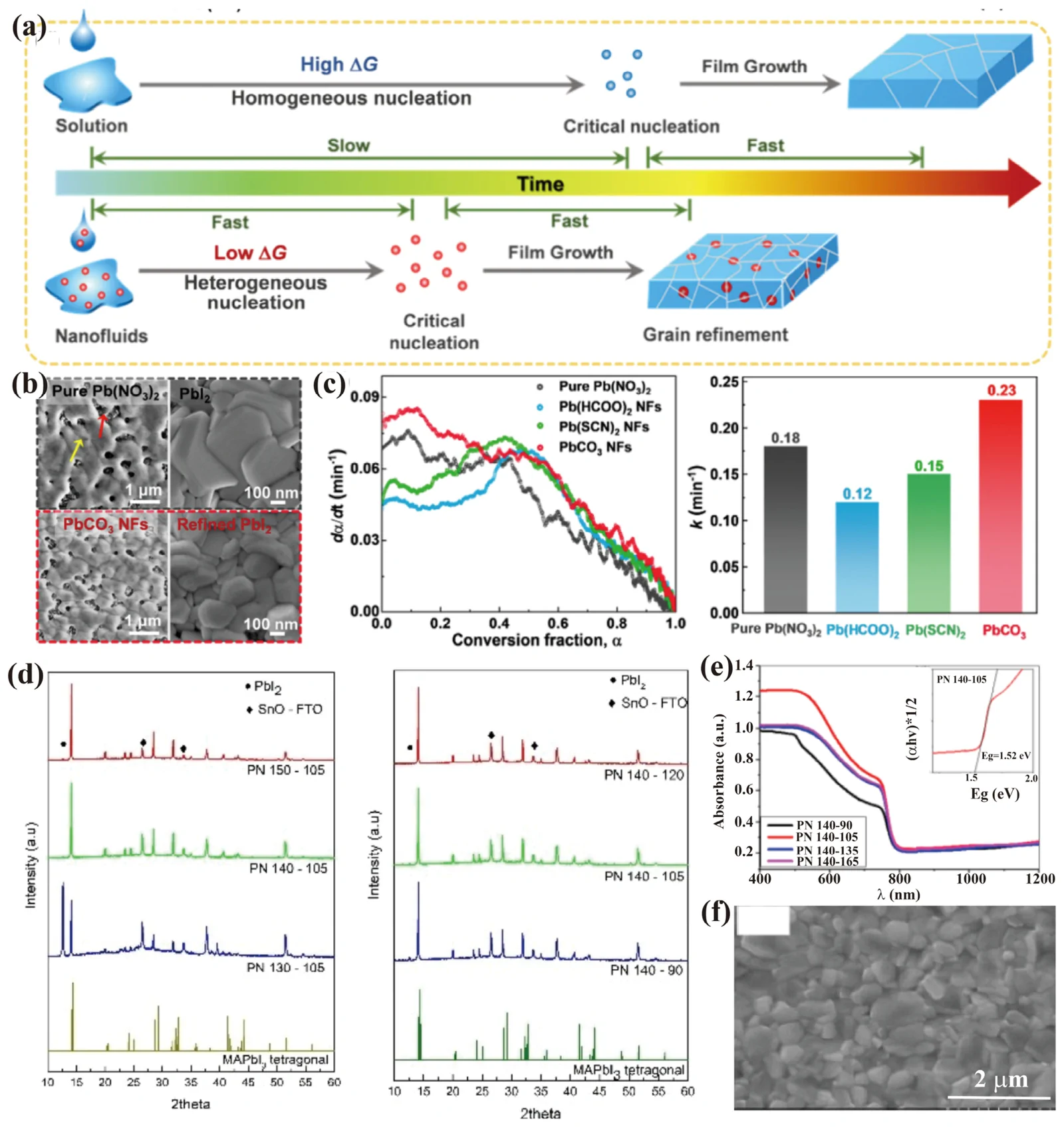
(**a**) Schematic diagram of the crystallization process of Pb(NO_3_)_2_ with and without adding nucleating seeds. (**b**) Pb(NO_3_)_2_ and PbI_2_ films prepared from pure Pb(NO_3_)_2_ solution and PbCO_3_ NFs, respectively. The red and yellow arrows indicate the mp-TiO_2_ substrate and Pb(NO_3_)_2_ film, respectively. (**c**) Reaction rate (dα/dt) as a function of conversion (α) and effective rate constants for various Pb(NO_3_)_2_ substrates [49] (reproduced from the ref. [49]; Copyright 2023, Royal Society of Chemistry) (**d**) XRD patterns of perovskite films prepared from Pb(NO_3_)_2_ (denoted as PN) at varying temperatures after 105 min of reaction. (**e**) UV-vis absorption spectra of MAPbI_3_ films formed at 140 °C from the PN precursor over different reaction durations. (**f**) SEM surface morphology of perovskite films derived from PbI_2_ at 140 °C for 105 min [52] (reproduced from ref. [52]; Copyright 2023, Wiley-VCH GmbH).

The conversion of individual Pb(NO_3_)_2_ grains also proceeds from the exterior toward the interior, making precursor grain size another important factor in conversion kinetics. Zhai et al. introduced halogen-free nanoparticles into the precursor solution to promote heterogeneous nucleation of Pb(NO_3_)_2_ (Figure 7a) [49]. The additional nucleation sites refined the Pb(NO_3_)_2_ grains and increased the accessible reaction area (Figure 7b), resulting in an approximately 1.28-fold increase in the conversion rate and a reduction in the apparent activation energy of about 600 J/mol (Figure 7c). Zhang et al. introduced a small amount of MAI into the aqueous precursor to generate nanoscale PbI_2_ seeds in situ [50]. These seeds modified Pb(NO_3_)_2_ crystallization and provided additional reaction sites during subsequent conversion, increasing the conversion rate by nearly 30% and lowering the apparent activation energy by approximately 700 J/mol. The accelerated conversion was accompanied by improved perovskite-film morphology. Hoang et al. further reported that PbF_2_ addition improved precursor-solution wettability and promoted more uniform Pb(NO_3_)_2_ nucleation, accelerating perovskite phase transformation by approximately 30% [51].

### 4.2. Conversion Process and External-Field Engineering

Prolonged exposure of Pb(NO_3_)_2_ precursor films to OAS solutions can cause excessive grain growth, morphological deterioration, and nonuniform conversion. Reducing the time required for complete transformation is therefore important for preserving film quality.

**Figure 8:**
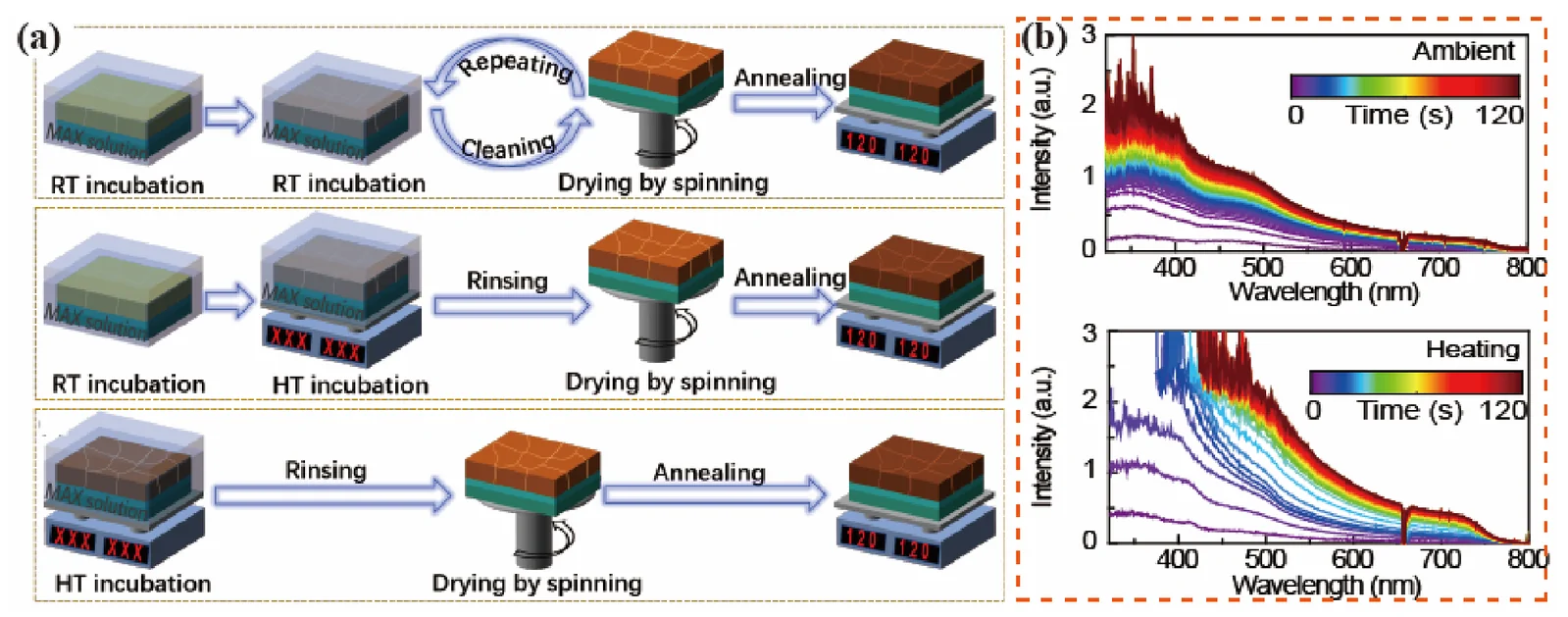
(**a**) The fabrication process of the perovskite film from Pb(NO_3_)_2_. (**b**) In-situ UV-vis absorption spectra of Pb(NO_3_)_2_ films immersed in OAS solution under ambient and heated conditions [57] (reproduced from ref. [57]; Copyright 2025, Elsevier).

Repeated short-cycle immersion has been used instead of prolonged continuous immersion to limit excessive grain growth while maintaining sufficient precursor conversion [53,54,55]. Pothiklang et al. further employed drop casting to deliver the OAS solution and found that relatively low MAI concentrations favored more complete conversion [56]. At higher MAI concentrations, rapid reaction near the film surface can restrict subsequent OAS penetration into deeper regions, resulting in nonuniform conversion and residual species. Nguyen et al. used MAI vapor to avoid direct contact between the precursor film and a liquid OAS solution [52]. The conversion increased with reaction temperature, whereas prolonged treatment at elevated temperature promoted partial decomposition of the formed perovskite to PbI_2_, as shown in Figure 7d–f. Dai et al. showed that thermal assistance promotes both OAS transport and the associated conversion reactions within Pb(NO_3_)_2_ precursor films [57]. The heat-assisted process accelerated perovskite formation and improved film crystallinity, while reducing residual stress and defect density (Figure 8a,b). Zhai et al. used light irradiation to regulate the conversion process and improve the morphology of water-based perovskite films [32]. They proposed that illumination modifies the interfacial charge distribution at the PbI_2_/OAS interface, facilitating halide accumulation and subsequent perovskite formation. Increasing the illumination intensity reduced the grain size and gradually suppressed film pinholes, leading to a device PCE of 23.74%.

### 4.3. Ionic and Compositional Regulation

The sequential conversion of aqueous Pb(NO_3_)_2_ films is fundamentally an ion-exchange and diffusion process. During incubation, OAS-derived cations and halide ions migrate into the precursor film, while nitrate-containing species diffuse outward. The conversion kinetics are therefore influenced by ionic size, solvation, coordination with Pb^2+^, concentration, and precursor microstructure.

The incorporation of Br^−^ or Cl^−^ has been reported to accelerate the conversion of Pb(NO_3_)_2_ precursor films and improve film morphology [58]. Their smaller effective ionic radii relative to I^−^ may reduce transport constraints, while their different interactions with Pb^2+^ can also alter ion-exchange and crystallization behavior [59]. The enhanced conversion should therefore be attributed to the combined effects of ionic transport and Pb-halide interactions rather than ionic size alone. At the same time, the halide composition of the final perovskite must be carefully controlled because excessive Br incorporation can induce light-induced phase segregation in mixed-halide perovskites [60,61]. The organic cation also influences the conversion behavior. FA^+^ (2.53 Å) has a larger effective ionic size than MA^+^ (2.17 Å) [62], and increasing the FA content generally increases the time required for complete conversion in the aqueous Pb(NO_3_)_2_ route [41]. This behavior is consistent with increased transport and structural constraints during incorporation of FA-containing species. Subsequent thermal annealing can promote redistribution and incorporation of residual FA-containing species into the perovskite lattice. The corresponding changes in film morphology highlight that compositional design in water-based systems must simultaneously consider the targeted perovskite composition and the transport kinetics required for complete precursor conversion.

The reported strategies can be further compared according to the primary process that they regulate during precursor formation and conversion. Representative approaches include solution modification, substrate regulation, pore and grain-size engineering, external-field assistance, and ionic/compositional regulation. Although these strategies act at different stages of fabrication, their effects can be compared in terms of OAS transport, conversion behavior, film quality, and device performance, as summarized in Table 2.

The comparison in Table 2 shows that these strategies act on different parts of the precursor-conversion process. Solution and substrate regulation primarily affect the formation of the initial Pb(NO_3_)_2_ film, whereas pore and grain-size engineering modify the transport pathways and distances for OAS. Thermal and light assistance promote the subsequent conversion process, while halide and organic-cation regulation alter ion transport and incorporation. Faster conversion does not necessarily produce a better perovskite film because excessively rapid surface conversion may restrict further OAS penetration. Suitable regulation should therefore promote conversion throughout the precursor film while maintaining controlled crystal growth.

## 5. Performance Comparison Between n-i-p and p-i-n Device Architectures

**Figure 9:**
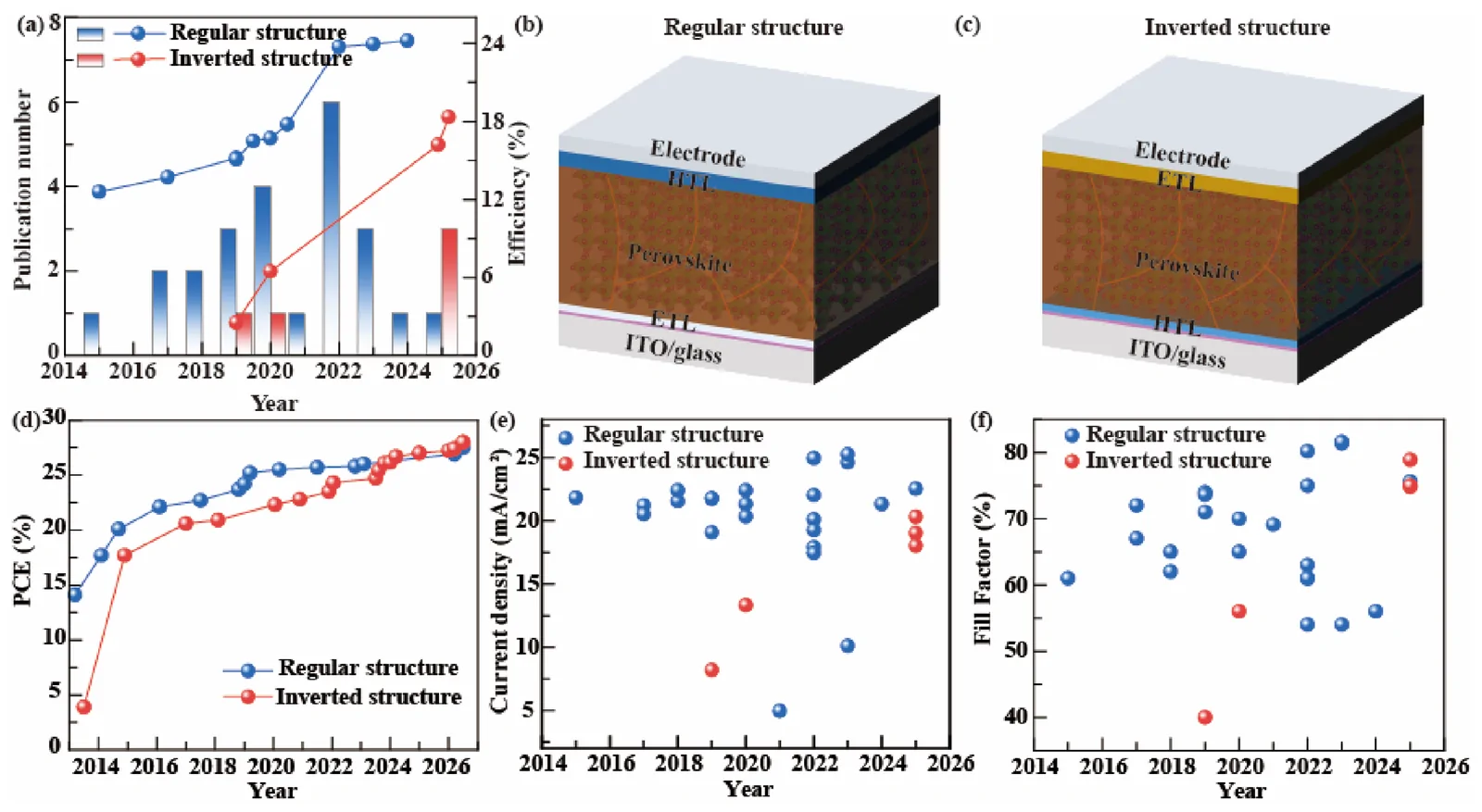
(**a**) Author-generated publication statistics and efficiency development of W-PSCs compiled by the authors from the cited literature. Author-generated schematic device configurations of (**b**) n-i-p and (**c**) p-i-n W-PSCs. (**d**) Efficiency development of conventionally processed PSCs compiled from published data. Literature-based distributions of (**e**) J_sc_ and (**f**) FF for reported W-PSCs [32,36,41,42,44,46,47,48,49,50,51,52,53,54,55,56,57,58,64,65,66,67,68,69,70,71,72,73].

Continuous improvements in precursor-film formation and conversion kinetics have increased the reported champion PCE of W-PSCs from approximately 12% to 24.14%, as summarized in Figure 9a [36,47]. Both regular (n-i-p) and inverted (p-i-n) architectures have been demonstrated for PSCs [63], as shown in Figure 9b,c. Among the studies summarized in this review, higher efficiencies have generally been obtained using regular architectures, whereas the reported performance of inverted W-PSCs remains lower. This trend differs from that observed in state-of-the-art organic-solvent-processed PSCs, as summarized in Figure 9d. As shown in Figure 9e,f, inverted W-PSCs generally exhibit lower J_sc_ and FF than their regular counterparts. These differences are associated with multiple factors, including precursor-film formation, absorber thickness, conversion completeness, crystallinity, interfacial recombination, and charge transport. The following discussion therefore focuses on how substrate-dependent precursor deposition and subsequent conversion may contribute to the performance gap between the two device architectures.

To enable a more direct comparison across reported W-PSCs, representative devices are further summarized in Table 3 with respect to precursor composition, substrate, conversion conditions, absorber thickness, device architecture, active area, photovoltaic parameters, and stability. The table is intended as a representative benchmark rather than an exhaustive collection because the parameters reported in different studies are not fully uniform. The comparison in Table 3 indicates that the reported photovoltaic performance varies with precursor composition, substrate, conversion procedure, absorber thickness, device architecture, and active area. These differences should therefore be considered when comparing device efficiencies across different studies. In particular, comparison between regular and inverted W-PSCs should account for differences in absorber thickness, conversion completeness, transport layers, and device area in addition to the nominal PCE.

**Figure 10:**
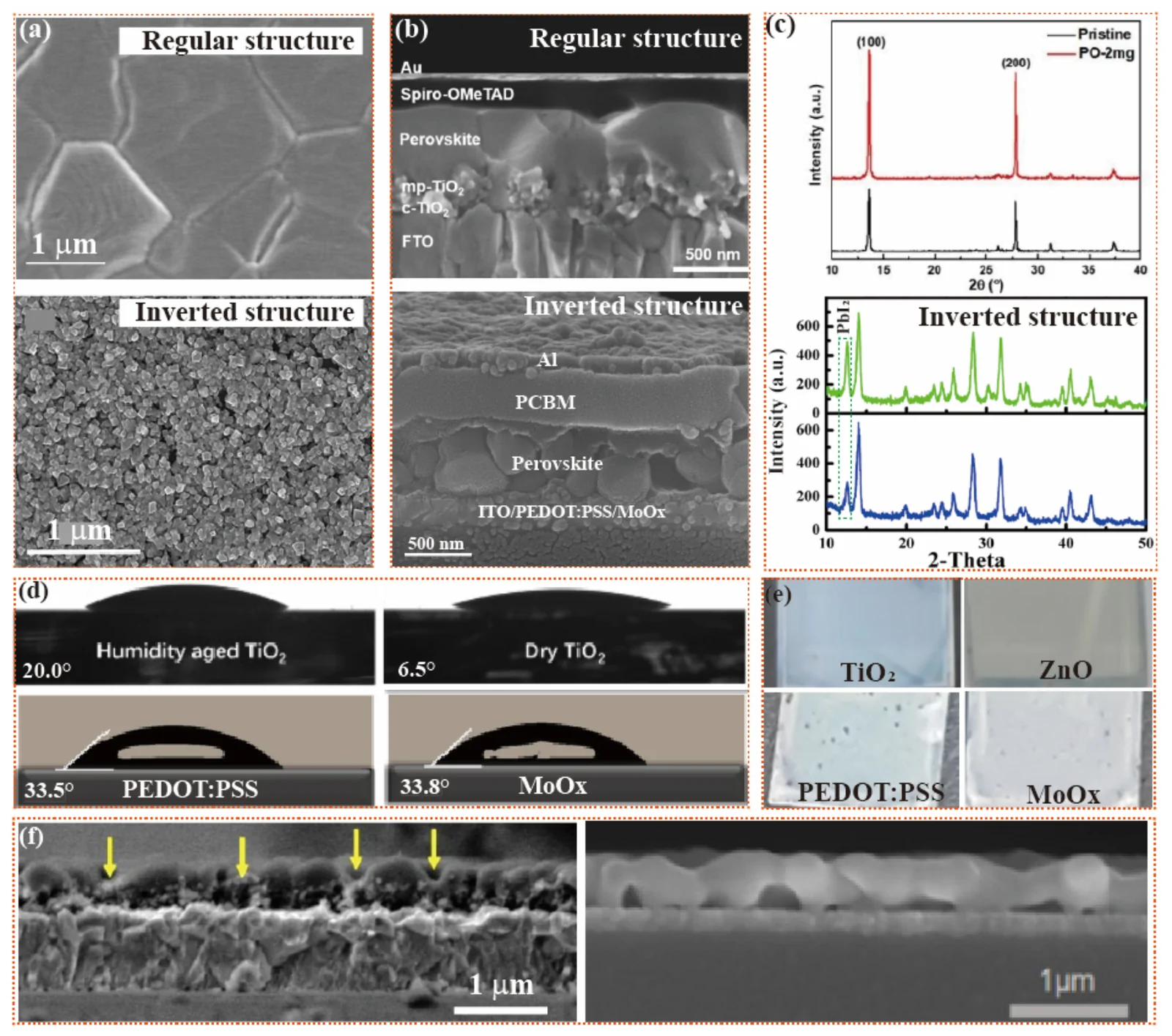
(**a**) Surface SEM and (**b**) Cross-sectional SEM images of perovskite films based on regular and inverted structures [32,47,67] (reproduced from ref. [32,47,67]; Copyright 2024, Royal Society of Chemistry. Copyright 2020, Wiley-VCH GmbH). (**c**) XRD of perovskite films for regular and inverted structures [32,67]. (**d**) The contact angle of the precursor solution on TiO_2_, PEDOT:PSS and MoO_x_ substrates [47,53,67]. (**e**) Optical photographs of the precursor film deposited on TiO_2_, ZnO, PEDOT:PSS and MoO_x_ substrates [47,53,67]. (**f**) Cross-sectional SEM images of precursor films deposited on TiO_2_ and PEDOT:PSS [32,42]. The yellow arrows indicate the pores in the film. (Reproduced from ref. [32,42,47,53,67]; Copyright 2024, Royal Society of Chemistry. Copyright 2020, Wiley-VCH GmbH. Copyright 2025, Multidisciplinary Digital Publishing Institute. Copyright 2018, Nature.)

The SEM images in Figure 10a,b show clear differences in the morphology of water-based perovskite films deposited on regular and inverted substrates [32,47,67]. Films prepared on regular substrates generally exhibit larger grains and more compact cross-sectional morphologies, whereas those formed on the investigated inverted substrates contain smaller stacked grains and rougher surfaces. Unpassivated grain boundaries and structural defects can introduce non-radiative recombination pathways and impede carrier transport. The absorber layers reported for the inverted structures are also generally thinner than those used in the regular devices. Reduced absorber thickness may contribute to lower optical absorption and J_sc_, although charge-collection efficiency and interfacial recombination must also be considered. XRD measurements further indicate differences in phase conversion between the two architectures. The regular-structure films show stronger perovskite diffraction features and relatively little residual PbI_2_, whereas noticeable PbI_2_ remains in inverted-structure films [32,67], as shown in Figure 10c. Because diffraction intensity is also affected by film thickness and preferred orientation, these observations should not be interpreted solely as differences in crystallinity. Nevertheless, together with the morphological evidence, these results suggest that incomplete precursor conversion may contribute to the poorer film quality observed in some inverted W-PSCs.

The morphology and conversion behavior of the final perovskite film are closely related to the structure of the initial Pb(NO_3_)_2_ layer. Figure 10d compares the contact angles of aqueous precursor solutions on TiO_2_, PEDOT:PSS, and MoO_x_ substrates [47,53,67]. The lower contact angle observed on TiO_2_ indicates more favorable spreading of the aqueous precursor under the reported conditions. This improved wetting facilitates formation of a more continuous Pb(NO_3_)_2_ layer, whereas poor wetting on some inverted substrates can promote nonuniform deposition and macroscopic pinholes, as shown in Figure 10e [47,53,67]. Cross-sectional SEM images further show substrate-dependent differences in Pb(NO_3_)_2_ grain structure and interfacial contact (Figure 10f) [32,42]. Importantly, the precursor films prepared on different substrates also exhibit different pore structures. Controlled and interconnected pores can serve as pathways for OAS transport during conversion, whereas large discontinuities or interfacial voids are detrimental because they may remain as structural defects after conversion. In several inverted structures, the precursor layer lacks well-connected through-thickness transport pathways while simultaneously showing poor interfacial coverage. Under these conditions, rapid formation of perovskite near the exposed surface can increase the resistance to further OAS penetration, leaving deeper precursor regions incompletely converted.

Taken together, the available studies suggest that substrate-dependent precursor-film formation is one of the major factors contributing to the performance difference between regular and inverted W-PSCs. Differences in wettability, interfacial interaction, precursor grain structure, and pore connectivity can influence both the initial Pb(NO_3_)_2_ morphology and the subsequent reaction–diffusion process. However, comparison across different reports should also consider differences in perovskite composition, absorber thickness, transport layers, processing conditions, device area, and measurement protocols. Further improvement of inverted W-PSCs therefore requires simultaneous optimization of aqueous-precursor deposition, through-thickness ion-transport pathways, conversion completeness, and buried-interface quality.

## 6. Conclusions and Outlook

Water-based perovskite fabrication offers an attractive route for reducing the dependence on hazardous polar aprotic solvents. Among the aqueous lead precursors investigated to date, Pb(NO_3_)_2_ has received particular attention because of its high water solubility and favorable ion-exchange characteristics. However, converting a Pb(NO_3_)_2_ precursor film into a compact and fully converted perovskite layer remains more complex than conventional solution crystallization. The process involves coupled ion exchange, OAS diffusion, nitrate removal, nucleation, and crystal growth, and these processes evolve continuously as the precursor film is transformed.

Recent progress shows that the microstructure of the Pb(NO_3_)_2_ precursor is particularly important. A suitable precursor film should combine high coverage with accessible and interconnected transport pathways. Such porosity facilitates penetration of OAS into the film interior, whereas excessive macroscopic pinholes or interfacial voids are detrimental to the final device. Regulation of substrate wettability, precursor nucleation, grain size, pore structure, temperature, external energy input, and ionic composition has therefore become central to improving conversion kinetics and final film quality.

Despite these advances, several challenges remain. First, the relationship between substrate surface properties and the early-stage nucleation of aqueous Pb(NO_3_)_2_ films requires further clarification, particularly for inverted device architectures. Improved control over substrate surface energy, surface potential, and interfacial chemistry may provide a route toward uniform precursor deposition on a wider range of charge-transport layers. Second, the reaction–diffusion process should be investigated using time-resolved structural and spectroscopic techniques to distinguish the contributions of interfacial reaction, ion transport, intermediate-phase formation, and crystal growth. Quantitative understanding of pore connectivity and effective diffusivity would also help establish a clearer relationship between precursor microstructure and conversion rate.

For scalable fabrication, aqueous precursor formulations must be compatible with large-area coating methods while maintaining uniform thickness and reproducible drying behavior. Additives and processing aids should be evaluated not only for their effects on efficiency but also for their influence on environmental impact, waste treatment, and long-term device stability. In addition, water-based processing does not eliminate the need for responsible Pb management. Recovery and treatment of Pb-containing aqueous waste should therefore be incorporated into future process design.

Finally, a comprehensive life-cycle assessment is needed to determine whether replacing conventional precursor solvents with aqueous systems leads to a meaningful reduction in the overall environmental footprint of PSC manufacturing. Such assessments should include precursor synthesis, solvent consumption, energy input, Pb recovery, device lifetime, and end-of-life recycling. Progress in these areas will help establish water-based perovskite photovoltaics as a more scalable and environmentally responsible manufacturing route.

## Figures and Tables

**Table 1:** Solubility of common lead salts in water.

Material	Molecular Formula	Solubility (mg/mL)	
		20 °C	100 °C
Lead iodide	PbI_2_	0.63	4.10
Lead bromide	PbBr_2_	9.70	47.5
Lead chloride	PbCl_2_	9.90	33.4
Lead carbonate	PbCO_3_	10^−7^	10^−7^
Lead oxide	PbO	1.7 × 10^−2^	5 × 10^−2^
Lead hydroxide	Pb(OH)_2_	1.5 × 10^−3^	5 × 10^−4^
Lead thiocyanate	Pb(SCN)_2_	0.46	2.04
Lead acetate	Pb(Ac)_2_	443	2000
Lead nitrate	Pb(NO_3_)_2_	565	1270

**Table 2:** Representative strategies for regulating precursor conversion in W-PSCs.

Representative Strategy	Method	Effect on Ion Transport/Conversion	Film Quality	Champion PCE	Ref.
Solution regulation	PO additive	Improved wetting and precursor formation	More uniform and compact films	24.14%	[47]
Substrate regulation	Temperature-modulated NiO_x_ treatment	More uniform precursor deposition	Improved coverage	18.34%	[44]
Film regulation	SDS additive	Enhanced OAS transport	Improved morphology and crystallinity	14.82%	[42]
Film regulation	Nanoparticles	Reduced precursor grain size and transport distance	Improved morphology	23.95%	[49]
External-field engineering	Thermal assistance	Accelerated OAS transport and conversion	Improved crystallinity and reduced stress	16.20%	[57]
External-field engineering	Light assistance	Accelerated conversion	Reduced pinholes	23.74%	[32]
Compositional regulation	Halide regulation	Accelerated ion exchange and conversion	Improved morphology	15.11%	[58]
Compositional regulation	Cation regulation	Higher FA^+^ content slows conversion	Composition-dependent morphology	16.50%	[41]

**Table 3:** Benchmark comparison of representative W-PSCs.

Aqueous Precursor	PSCs Structure	Conversion Conditions	Thickness (nm)	Area (cm^2^)	Voc (V)	J_sc_ (mA/cm^2^)	FF (%)	PCE (%)	Stability	Ref.
Pb(NO_3_)_2_/H_2_O	c-TiO_2_/mp-TiO_2_/MAPbI_3_/Spiro-OMeTAD/Au	soaking; RT; ~1000 s	NR	0.088	0.94	21.81	61	12.58	NR	[36]
Pb(NO_3_)_2_/EtOH/H_2_O	c-TiO_2_/mp-TiO_2_/ZnO/MAPbI_3_/Spiro-OMeTAD/Ag	repeated dipping; RT; 4 cycles	550	0.045	0.93	21.53	62	12.41	NR	[53]
Pb(NO_3_)_2_/H_2_O	c-TiO_2_/mp-TiO_2_/MAPbX_3_/Spiro-OMeTAD/Au	immersion; RT; 500 s	400	0.1	1.07	19.07	74	15.11	93% (55 days, 1 sun)	[58]
Pb(NO_3_)_2_/H_2_O	PEDOT:PSS/MAPbI_3_/PCBM/Au	RT; spin-coating	NR	NR	0.78	8.19	40	2.53	NR	[56]
Pb(NO_3_)_2_/H_2_O	SnO_2_/BK-TiO_2_/FAxMA_x_Pb(I_0.9_Br_0.1_)_3_/Spiro-OMeTAD/Au	repeated dipping; RT; 500 s	500	0.06	1.03	21.77	74	16.50	75.8% (50 days, 25 °C, 10~25% RH)	[41]
Pb(NO_3_)_2_/H_2_O	c-TiO_2_/mp-TiO_2_/MAPbI_3_/Spiro-OMeTAD/Au	repeated dipping; RT; 500 s	NR	NR	1.08	22.4	70	16.7	NR	[66]
Pb(NO_3_)_2_/H_2_O	PEDOT:PSS/MoO_x_/MAPbI_3_/PCBM/Au	soaking; RT	417	0.045	0.86	13.33	56	6.46	NR	[67]
Pb(NO_3_)_2_/H_2_O	c-TiO_2_/mp-TiO_2_/FAPbI_3_/Spiro-OMeTAD/Au	soaking; RT; light-assisted conversion	715	0.0812	1.19	24.94	80	23.74	94% (60 days, 30%RH, dark)	[32]
Pb(NO_3_)_2_ + MAI/H_2_O	SnO_2_/CsMAFAPbI_3_/Spiro-OMeTAD/Au	spray-coating	420	0.16	1.00	22.01	75	16.53	89% (400 h, 20–30% RH, 25–28 °C)	[50]
Pb(NO_3_)_2_ + PbCO_3_/H_2_O	c-TiO_2_/mp-TiO_2_/FAPbI_3_/Spiro-OMeTAD/Au	repeated dipping; RT; 500 s	702	0.0812	1.18	24.95	82	23.95	92.5% (800 h, N_2_, 85 °C)	[49]
Pb(NO_3_)_2_ + PO/H_2_O	c-TiO_2_/mp-TiO_2_/FAPbI_3_/Spiro-OMeTAD/Au	repeated dipping; RT; 500 s	NR	0.0812	1.17	25.24	82	24.14	93.1% (800 h, N_2_, 85 °C)	[47]
Pb(NO_3_)_2_ + PbF_2_/H_2_O	SnO_2_/CsMAFAPbI_3_/Spiro-OMeTAD/Au	repeated dipping; RT	326	0.09	1.08	22.17	76	18.10	39.3% (120 h, 60%RH, 30 °C)	[51]
Pb(NO_3_)_2_/H_2_O	PEDOT:PSS/MAPbI_3_/PCBM/BCP/Ag	Soaking; 50 °C; ~180 s	347	0.0625	1.04	19.01	82	16.20	94.76% (2000 h, N_2_)	[57]
Pb(NO_3_)_2_ + SDS/H_2_O	PEDOT:PSS/MAPbI_3_/PCBM/BCP/Ag	Soaking; 50 °C; 3 min	467	0.0625	1.04	18.02	79	14.82	90% (800 h, N_2_)	[42]
Pb(NO_3_)_2_/H_2_O	NiO_x_/FAPbI_3_/PCBM/BCP/Ag	Soaking; 50 °C; ~180 s	486	0.0625	1.03	22.58	79	18.34	90% (350 h, 85 °C, N_2_)	[44]

Conversion conditions are summarized from the original reports; RT denotes room temperature and NR denotes not reported.

## Data Availability

No new data were created or analyzed in this study. Data sharing is not applicable to this article.

## Abbreviations

The following abbreviations are used in this manuscript:

PSCs	Perovskite solar cells
W-PSCs	Water-based perovskite solar cells
OAS	Organic ammonium salts
